# Multi-level multi-view network based on structural contrastive learning for scRNA-seq data clustering

**DOI:** 10.1093/bib/bbae562

**Published:** 2024-11-04

**Authors:** Zhenqiu Shu, Min Xia, Kaiwen Tan, Yongbing Zhang, Zhengtao Yu

**Affiliations:** Faculty of Information Engineering and Automation, Kunming University of Science and Technology, Chenggong, 650500, Yunnan, China; Faculty of Information Engineering and Automation, Kunming University of Science and Technology, Chenggong, 650500, Yunnan, China; Faculty of Information Engineering and Automation, Kunming University of Science and Technology, Chenggong, 650500, Yunnan, China; Faculty of Information Engineering and Automation, Kunming University of Science and Technology, Chenggong, 650500, Yunnan, China; Faculty of Information Engineering and Automation, Kunming University of Science and Technology, Chenggong, 650500, Yunnan, China

**Keywords:** multi-level, multi-view, shallow views, deep views, graph laplacian filter, contrastive clustering

## Abstract

Clustering plays a crucial role in analyzing scRNA-seq data and has been widely used in studying cellular distribution over the past few years. However, the high dimensionality and complexity of scRNA-seq data pose significant challenges to achieving accurate clustering from a singular perspective. To address these challenges, we propose a novel approach, called multi-level multi-view network based on structural consistency contrastive learning (scMMN), for scRNA-seq data clustering. Firstly, the proposed method constructs shallow views through the $k$-nearest neighbor ($k$NN) and diffusion mapping (DM) algorithms, and then deep views are generated by utilizing the graph Laplacian filters. These deep multi-view data serve as the input for representation learning. To improve the clustering performance of scRNA-seq data, contrastive learning is introduced to enhance the discrimination ability of our network. Specifically, we construct a group contrastive loss for representation features and a structural consistency contrastive loss for structural relationships. Extensive experiments on eight real scRNA-seq datasets show that the proposed method outperforms other state-of-the-art methods in scRNA-seq data clustering tasks. Our source code has already been available at https://github.com/szq0816/scMMN.

## Introduction

The development of single-cell sequencing (scRNA-seq) technology has significantly enhanced our understanding of cellular heterogeneity and complex disease mechanisms [1, 2]. Concurrently, scRNA-seq data has played a pivotal role in elucidating epigenetic variability within cellular systems. Furthermore, it offers a more targeted approach compared to batch sequencing. However, it is a challenging task to identify new cell types and group similar cells due to the high-dimensional and complex characteristics of scRNA-seq data. Thus, clustering can identify cells with similar gene expression, which is a pivotal step in scRNA-seq data analysis.

To obtain satisfactory clustering performance, various scRNA-seq data clustering methods have been proposed over the past few years [3–6]. Generally speaking, one important step in scRNA-seq data clustering is to extract its feature Information. Therefore, traditional methods, such as principal components analysis (PCA), nonnegative matrix factorization (NMF), sparse representation, and low-rank representation (LRR), were used to extract the features of scRNA-seq data. Schaub et al. [7] employed PCA to project scRNA-seq data into low-dimensional space, and then integrated the results from multiple clustering algorithms to construct a consensus matrix. Finally, they performed clustering operations on this consensus matrix. Therefore, it can effectively improve the robustness of the model. rssNMF [8] adopts NMF to decompose the gene expression matrix by imposing a graph regularization constraint, which can enhance the robustness of the model and achieve good clustering results. DLNLRR [9] constructs the subspaces of high-dimensional scRNA-seq data to realize dictionary learning and LRR learning at the same time. Then it directly clustering the samples on the low-rank matrix instead of spectral clustering. NLRRC [10] utilizes LRR to reduce the dimensionality of cells and then employs NMF to obtain their representation information. SPARC [11] proposes a new similarity measure strategy, which assesses the similarity between cells by the sparse representation of each cell concerning other cells. However, due to the highly nonlinear and complex characteristics of scRNA-seq data, traditional methods have limitations in learning inherent latent structures of cells [12].

In contrast to traditional methods, deep learning employs numerous trainable parameters to steer model training, adeptly capturing both linear and nonlinear cell relationships [13]. Therefore, various deep learning-based methods have been developed for clustering scRNA-seq data [14–17]. Among them, the autoencoder-based scRNA-seq data clustering methods [18–22] have shown promising performances in real applications. Specifically, the scRNA-seq data are mapped to the bottleneck layer through an encoder network to produce latent features for clustering. Then the decoder network reconstructs the bottleneck layer data and introduces the reconstruction loss to accurately reconstruct the data. sc-INDC [23] maximizes the mutual information between the input and output representations of the autoencoders, aiming to improve its efficiency. scMCKC [24] learns the latent representation of cells by autoencoders and then introduces compactness constraints to emphasize the closeness between cells. Finally, it utilizes the soft $k$-means algorithm to enhance the clustering accuracy of cells. scMAE [25] first perturbs gene data and subsequently employs a masked autoencoder to reconstruct the original data. Therefore, it can significantly reduce the noise impact in clustering. CAKE [26] implements the feature representation of cell data in two steps. Firstly, the coarse representation of cells can be learned through contrastive learning. Secondly, it refines the representation with precision using knowledge distillation technology.

Several scRNA-seq data clustering methods based on graph convolutional networks (GCNs) have also been proposed in recent years [27–30]. GCNs update each node by aggregating information from its neighboring nodes, enabling the model to learn feature representations and structural relationships between cells [31]. scMoGNN [32] constructs adjacency matrices based on cell modalities and subsequently feeds them into GCNs to train the model. Therefore, it effectively captures higher-order relationships between cells and modalities. Different from most GCN-based scRNA-seq data clustering methods that use cell-cell adjacency matrices as the input, graph-sc [33] feeds cell-gene graphs into GCNs, enhancing the flexibility for clustering scRNA-seq data. To further improve the clustering performance of GCNs, scDGDC [34] employs two-stream GCNs to learn the feature representation and structural relationships of cells. Moreover, it imposes triple constraints to solve the over-smoothing problem of GCNs. Additionally, scVGAE [35] combines GCNs and ZINB loss to realize the imputation of scRNA-seq data values, which solves the problem of dropout events in scRNA-seq data. scASDC [36] combines GCN and attention fusion mechanism, so that the representation information and structural information of each layer in GCNs can be effectively combined.

However, the aforementioned scRNA-seq data clustering methods are founded on a single view, potentially failing to accurately capture the feature representation of the data [37]. Therefore, several multi-view scRNA-seq data clustering methods [38–40] have been proposed recently. ScCCL [41] employs the dropout network to randomly delete cell expression genes and introduce Gaussian noise to construct multi-view scRNA-seq data. In addition, it incorporates contrastive loss to guide model training by minimizing the distance between cells of the same class and maximizing the distance between cells from different classes. scGAC [42] constructs multi-view data by extracting the expression data of the top $k$ genes. Then it learns the linear and nonlinear relationships between cells and generates a similarity matrix through GAE, thereby effectively guiding the model training. However, the above clustering methods adopt the shallow methods to construct multi-view data. Therefore, they cannot fully utilize the complementarity of multi-view scRNA-seq data to fully explore its characteristics.

In this paper, we introduce a multi-level multi-view network based on structural consistency contrastive clustering (scMMN), for clustering scRNA-seq data from the perspective of multi-level and multi-view. Firstly, two shallow representations of cells are constructed by constructing two different adjacency matrices. Then four deep views are generated using the graph Laplacian filters (GLFs). In addition, our scMMN method employs a multi-level contrastive learning strategy to guide the model training. Specifically, we perform contrastive learning from the perspectives of two levels of features and structure relationships, thereby ensuring consistency in both cell features and structure. Finally, we construct a feature fusion and clustering module to obtain the final clustering results. Extensive experimental results have demonstrated the advantage of the proposed method in scRNA-seq data clustering applications.

The main contributions of this work can be summarized as follows:

(1) We propose a novel multi-level multi-view contrastive clustering framework for scRNA-seq data clustering. It generates the adjacency matrix of cells by a common composition, called shallow view. Then the graph Laplacian filters are applied to construct deep views. This multi-level multi-view strategy facilitates the acquisition of more accurate feature representations of cells from multiple perspectives, thereby benefiting subsequent representation learning and clustering.
(2) A feature and structural contrastive learning strategy is introduced from multi-view aspects to guide model training. On the one hand, the proposed method employs a group contrastive learning strategy on the learned features to learn common features from different views. on the other hand, we construct a structural consistency contrast learning to fully explore the consistent structure relationship between cells.
(3) We conduct comprehensive experiments on eight scRNA-seq datasets to evaluate the performance of the proposed scMMN method. The experimental results indicate the superiority of the proposed method over other state-of-the-art scRNA-seq data clustering methods.

The remainder of this paper is organized as follows: Section 2 introduces the proposed scMMN method. Section 3 presents our experiments and results analysis. Section 4 concludes our paper.

## Methods

**Figure 1:**
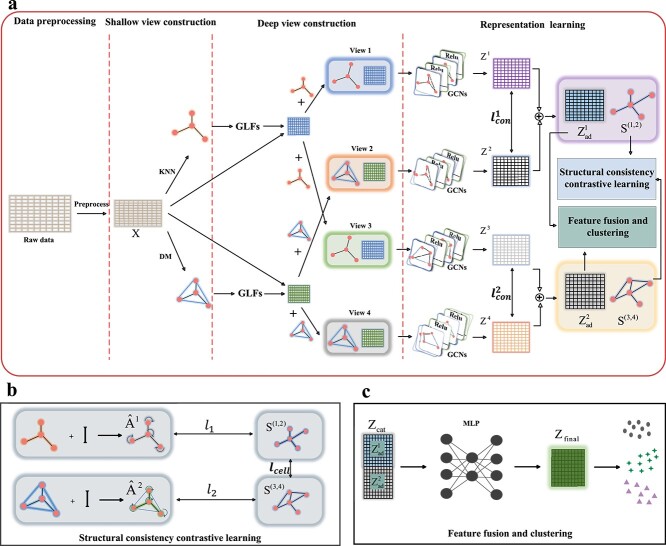
Overview of the proposed scMMN method. (a) The raw scRNA-seq data are preprocessed in the data preprocessing module. In the multi-view construction module, shallow views are constructed using the two different graph learning methods, and deep features are constructed using several GLFs. Afterwards, the learned views are fed into the representation learning module. This module initially learns latent representations through GCNs, and then guides model training through feature and structural contrastive learning. (b) The structural consistency contrastive learning module mainly computes the contrastive loss between the self-loop adjacency matrix $\hat{A}^{i}$ and the learned adjacency matrix $S_{ij}^{(a,b)}$. (c) The feature fusion module employs MLP to fuse multi-view data and learn a common representation for the final clustering.

In this paper, we propose a novel multi-level multi-view network based on structural consistency contrastive clustering, called scMMN, for scRNA-seq data clustering. It mainly includes four modules: data reprocessing, shallow view construction, deep view construction, and representation learning, as shown in Fig. 1. The data preprocessing module aims to normalize the gene expression matrix and extract the highly expressed genes. The shallow view construction module constructs two similarity matrices of cells using the $k$NN and DM algorithms and then derives the shallow representation of cells. The deep view construction module first constructs the graph Laplacian filters (GLFs) and then uses them to learn the deep representation of cells. The representation learning module learns the latent representation of cells through GCNs, and then performs contrastive learning on the learned features, so that the features are as similar as possible between different views. Besides, a structural consistency contrastive learning block is designed to facilitate the feature learning of multi-view data, ensuring structural consistency across different views. Finally, we feed the representations of different views into the feature fusion and clustering block to obtain the final results.

### Data preprocessing

To improve the robustness of the clustering models, we need to preprocess raw scRNA-seq data. The detailed steps can be described as follows: Firstly, we filter out a minimum number of genes and cells, removing unexpressed genes and cells with no gene expression across all cells. Next, the normalization process is conducted. Finally, the top 2000 or 3000 highly expressed genes are reserved according to the characteristics of the scRNA-seq dataset. The preprocessing of scRNA-seq data is performed by utilizing the Scanpy package [43].

### Shallow view construction

In this module, we aim to construct two graph structures using the $k$NN and DM algorithms. Specifically, the KNN algorithm constructs the gene expression matrix from scRNA-seq data and computes the distance between cell nodes in Euclidean space. Meanwhile, DM seeks to convert Euclidean distance into non-Euclidean distance through the Gaussian kernel function. Therefore, we can effectively capture the linear and nonlinear structures inherent in scRNA-seq data by utilizing the $k$NN and DM algorithms. Next, we will introduce the construction process of the shallow views.

Suppose the gene expression matrix is expressed as $X\in \ R^{n\times m}$, where $n$ represents the number of preprocessed cells, and $m$ is the number of genes. The graph structure of cells is shown as $G=(A, X)$, where $A\in \ R^{n\times n}$ denotes the adjacency matrix between cells. The edges between cells are represented as $V=(v_{1},v_{2},......,v_{n})$. Firstly, the Euclidean space distance between cell nodes is calculated, and the distance matrix $D\in \ R^{n\times n}$ is derived. Therefore, we can easily obtain the top $k$ nearest neighbors of each cell, and then set the edge weight value to 1 for its neighboring cells and 0 for other cells. Thus, we can construct the affinity matrix denoted $A_{KNN}$ for the graph structure using the $k$NN algorithm as outlined previously. Thus, we have


(1) \begin{align*}& \begin{aligned} A_{KNN}=\begin{cases}1,\ \ &x\in KNN(x_{i}) \\0,\ \ &otherwise\end{cases}. \end{aligned}\end{align*}

To explore the nonlinear structure of scRNA-seq data, we leverage the DM algorithm to construct the graph structure within a non-Euclidean space by utilizing the Gaussian kernel function. The distances within the non-Euclidean space using the DM algorithm are calculated as follows:


(2) \begin{align*}& \begin{aligned} \bar{d_{ij}}=\frac{1}{2}\left(e^{(-\frac{d_{ij}}{{2\partial_{k}(x_{i})}^{2}})}+e^{(-\frac{d_{ij}}{{2\partial_{k}(x_{j})}^{2}})}\right), \end{aligned}\end{align*}

where $\partial _{k}(x_{i})$ represents the bandwidth of the Gauss kernel function. This bandwidth is determined by each cell and its neighbors. The weight values of the first $k$ nearest neighbor cells are set to 1, and the rest are set to 0. Then the graph structure $A_{DM}$ based on the DM algorithm can be constructed.

The adjacency matrix obtained by $k$NN algorithm and DM algorithm is not the same. Specifically, the number of adjacency matrix edges constructed by the DM algorithm is more than the adjacency matrix constructed by $k$NN. The reason is as follows. In $k$NN algorithm, the top $k$ nearest points are selected as neighbor nodes, and only the Euclidean distance between nodes is considered, without considering the distribution distance between nodes. When the physical distance between two nodes is far, the $k$NN algorithm judges that there is no connection between them. DM algorithm is a nonlinear algorithm, which determines whether a node is a neighbor node by its distribution information. That is, if two nodes are far apart in physical space, they can still be connected.

### Deep view construction

In this module, we propose a novel view construction method to construct deep views. We represent the diagonal matrix as $C =\ diag(c_{1},c_{2},......,c_{n})\in R^{n\times n}$, where $c_{i}=\sum _{(v_{i},v_{j})} a_{ij}$. The graph Laplace matrix can be expressed as $L=C\ -\ A$, and thus the normalized graph Laplacian matrix is expressed as $\hat{L}=I-{\hat{C}}^{(-\frac{1}{2})}\hat{A}{\hat{C}}^{(-\frac{1}{2})}$, where $\hat{A}=A+I$, and $\hat{C}$ is a diagonal matrix of $\hat{A}$. Firstly, we introduce the graph Laplace filter (GLF) $H$ as follows:


(3) \begin{align*}& \begin{aligned} H=I-\hat{L}. \end{aligned}\end{align*}

To increase the diversity between views, we stack $t$-layer filters and obtain the final feature matrix as follows:


(4) \begin{align*}& \begin{aligned} F=\left(\prod_{i\ =1}^{t}H\right)X=H^{t}X, \end{aligned}\end{align*}

where $H^{t}$ represents the stacked $t-$layer GLFs.

In the previous steps, we construct two adjacency matrices, namely $A_{KNN}$ and $A_{DM}$. Thus, we can obtain two normalized graph Laplacian matrices ${\hat{L}}_{KNN}$ and ${\hat{L}}_{KNN}$. According to Eq.(3), we can construct two different GLFs, namely $H_{KNN}$ and $H_{DM}$. Therefore, two different deep view features, $F_{KNN}$ and $F_{DM}$, are learned by stacking $t$ layer GLFs. We combine the adjacency matrix constructed in the shallow views with the features generated in this part to generate four different views, such as $G_{1} = (A_ {KNN}, F_ {KNN}) $, $G_{2} = (A_ {KNN}, F_ {DM}) $, $G_{3} = (A_ {DM}, F_ {KNN}) $, and $G_{4} = (A_ {DM}, F_ {DM}) $. Based on the different ways used to construct shallow views, we categorize the aforementioned four views into two groups: Group One, comprising $G_{1}$ and $G_{2}$, and Group Two, consisting of $G_{3}$ and $G_{4}$, to facilitate subsequent group contrastive learning.

### Representation learning

#### Latent feature learning in GCNs

In the representation learning module, we adopt GCNs to learn the features from four different views. Here, we denote the parameters of GCNs as $f_\theta $, where $\theta $ represents a matrix consisting of $W_{1}$ and $W_{2}$. Given a graph structure $G=(A, X)$, the representation learning for cells within two-layer GCNs can be expressed as follows:


(5) \begin{align*}& \begin{aligned} f_\theta\left(A,X\ \right)=softmax\left({\hat{D}}^{\left(-\frac{1}{2}\right)}\hat{A}{\hat{D}}^{\left(-\frac{1}{2}\right)}\right)\sigma \\ (({\hat{D}}^{(-\frac{1}{2})}\hat{A}{\hat{D}}^{(-\frac{1}{2})})XW_{1})W_{2}), \end{aligned}\end{align*}

where $\sigma $ represents an activation function. Here, we adopt $ReLU$ as the activation function in Eq. (5).

Specifically, we feed the four views learned from deep view construction into GCNs to obtain four latent representations, i.e. $Z^{1}$, $Z^{2}$, $Z^{3}$, and $Z^{4}$. Then the learned feature representations are normalized with $l_{2}-$norm. Thus, we have


(6) \begin{align*} & \begin{aligned} Z^{1}=\frac{Z^{1}}{\left\|Z^{1}\right\|_{2}}, \end{aligned} \end{align*}

(7) \begin{align*} & \begin{aligned} Z^{2}=\frac{Z^{2}}{\left\|Z^{2}\right\|_{2}}, \end{aligned} \end{align*}

(8) \begin{align*} & \begin{aligned} Z^{3}=\frac{Z^{3}}{\left\|Z^{3}\right\|_{2}}, \end{aligned} \end{align*}

(9) \begin{align*} & \begin{aligned} Z^{4}=\frac{Z^{4}}{\left\|Z^{4}\right\|_{2}}. \end{aligned} \end{align*}

Following the grouping strategy within the deep view construction module, the four potential features can be divided into two groups: $Z^{1}$ and $Z^{2}$ forming one group, $Z^{3}$ and $Z^{4}$ constituting another group. To obtain more accurate clustering results, we require that cells belonging to the same cluster are close to each other and cells from different clusters are further apart. To achieve this goal, group contrastive learning is proposed in our method. Suppose that the number of clusters on the dataset is $c$. The group contrastive loss functions for these two group features are defined as follows:


(10) \begin{align*} & \begin{aligned} l_{con}^{1} &=\frac{1}{2 c} \sum_{i=1}^{c}\left[l\left(Z_{, i}^{1}, Z_{, i}^{2}\right)+l\left(Z_{, i}^{2}, Z_{, i}^{1}\right)\right], \end{aligned} \end{align*}

(11) \begin{align*} & \begin{aligned} l_{con}^{2} &=\frac{1}{2 c} \sum_{i=1}^{c}\left[l\left(Z_{, i}^{3}, Z_{, i}^{4}\right)+l\left(Z_{, i}^{4}, Z_{, i}^{3}\right)\right], \end{aligned} \end{align*}

where


(12) \begin{align*} & \begin{aligned} &l\left(Z_{, i}^{a}, Z_{, i}^{b}\right)=-\log \frac{e^{\left(\varphi\left(Z_{, i}^{a}, Z_{, i}^{b}\right) / \tau\right)}}{\sum_{j=1, j \neq i}^{c} e^{\left(\varphi\left(Z_{, i}^{a}, Z_{, j}^{a}\right) / \tau\right)}+\sum_{j=1}^{c} e^{\left(\varphi\left(Z_{, i}^{a}, Z_{j}^{b}\right) / \tau\right)}}, \end{aligned} \end{align*}

(13) \begin{align*} & \begin{aligned} \varphi(a, b)=\frac{a \cdot b^{T}}{\|a\| \cdot\|b\|}, \end{aligned} \end{align*}

where $\tau $ is the temperature coefficient, and $\varphi \left (\cdot , \cdot \right )$ represents the cosine similarity of two sets of features.

We aggregate these two groups of feature contrast losses to derive the group contrast loss function as follows:


(14) \begin{align*}& \begin{aligned} l_{con}=l_{con}^{1}+l_{con}^{2}. \end{aligned}\end{align*}

#### Structural consistency contrastive learning

To learn the structural consistency between intersecting views, we propose a novel contrastive loss function based on neighbor cells in our proposed method. The similarity matrix between the cross-views can be constructed as follows:


(15) \begin{align*} & \begin{aligned} S_{ij}^{(1,2)}=Z_{i}^{1}\cdot\left(Z_{j}^{2}\right)^{T},\ \forall\ i,j\in[1,n], \end{aligned} \end{align*}

(16) \begin{align*} & \begin{aligned} S_{ij}^{(3,4)}=Z_{i}^{3}\cdot\left(Z_{j}^{4}\right)^{T},\ \forall\ i,j\in[1,n], \end{aligned} \end{align*}

where $S_{ij}^{(a,b)}$ represents the similarity between cell $i$ in the $a-th$ view and cell $j$ in the $b-th$ view.

After obtaining the similar matrix of cross-view data, we calculate the contrastive loss between the similarity matrix and the self-ring adjacency matrix. To maintain the original relationship between cells and emphasize the self-similarity of cells themselves, we can derive the self-ring adjacency matrices as follows:


(17) \begin{align*} & \begin{aligned} {\hat{A}}^{1}=A_{KNN}+\boldsymbol{I}, \end{aligned} \end{align*}

(18) \begin{align*} & \begin{aligned} {\hat{A}}^{2}=A_{DM}+\boldsymbol{I}, \end{aligned} \end{align*}

Then we compute the contrastive loss between the similarity matrix and the self-ring adjacency matrix as follows:


(19) \begin{align*} & \begin{aligned} l_{1} & =\frac{1}{n^{2}}\sum\left(S^{\left(1,2\right)}-{\hat{A}}^{1}\right)\ \\ &=\frac{1}{n^{2}}\left(\sum_{i}\sum_{j}\beth_{ij}^{1}\left(S_{ij}^{\left(1,2\right)}-1\right)^{2}+ \sum_{i}\sum_{j}\beth_{ij}^{0}{S_{ij}^{\left(1,2\right)}}^{2}\right), \end{aligned} \end{align*}

(20) \begin{align*} & \begin{aligned} l_{2} &=\frac{1}{n^{2}}\sum\left(S^{\left(2,3\right)}-{\hat{A}}^{2}\right) \\ &=\frac{1}{n^{2}}\left(\sum_{i}\sum_{j}\beth_{ij}^{1}\left(S_{ij}^{\left(3,4\right)}-1\right)^{2}+\sum_{i}\sum_{j}\beth_{ij}^{0}{S_{ij}^{\left(3,4\right)}}^{2}\right), \end{aligned} \end{align*}

where $\beth _{ij}^{1}$ means ${\hat{A}}_{ij}=1$, and $\beth _{ij}^{0}$ means ${\hat{A}}_{ij}=0$.

We consider the neighboring nodes of cells in different views between groups as positive samples, and cells with non-neighboring nodes as negative samples. The purpose of Eqs. 19,20 is to make the distance between positive samples closer and the distance between negative samples farther.

By integrating Eq.(19) and Eq.(20), the structural consistency loss can be written as follows:


(21) \begin{align*}& \begin{aligned} l_{s}=l_{1}+l_{2}. \end{aligned}\end{align*}

For the similarity matrices $S_{ij}^{\left (1,2\right )}$ and $S_{ij}^{\left (3,4\right )}$ between different views, we aim to align them as closely as possible. Therefore, we perform the contrastive loss between $S_{ij}^{\left (1,2\right )}$ and $S_{ij}^{\left (3,4\right )}$ as follows:


(22) \begin{align*}& \begin{aligned} l_{cell}=l\left(S^{\left(1,2\right)},S^{\left(3,4\right)}\right)+l\left(S^{\left(3,4\right)},S^{\left(1,2\right)}\right). \end{aligned}\end{align*}

In summary, the loss function of the proposed method is given as follows:


(23) \begin{align*}& \begin{aligned} loss={\alpha l}_{s}+\beta\ l_{con}+\gamma\ l_{cell}, \end{aligned}\end{align*}

where $\alpha $, $\beta $, and $\gamma $ represent hyperparameters.

#### Feature fusion and clustering

After the feature representation learning module, we can obtain four latent feature representations. To be able to cluster cells accurately, we need to fuse these four features by utilizing a two-step fusion strategy. Specifically, we conduct intra-group fusion followed by inter-group fusion. To learn more comprehensive fusion features, we feed the intra-group fusion features into an MLP, thus acquiring the common representation of cells between different groups.

To be specific, we first perform inter-group fusion on two sets of views to obtain the preliminary fused features as follows:


(24) \begin{align*} & \begin{aligned} {Z_{ad}^{1}= \big(Z}^{1}+Z^{2}\big)/2, \end{aligned} \end{align*}

(25) \begin{align*} & \begin{aligned} {Z_{ad}^{2}= \big(Z}^{3}+Z^{4} \big)/2. \end{aligned} \end{align*}

Next, we perform intra-group fusion on $Z_{ad}^{1}$ and $Z_{ad}^{2}$ as follows:


(26) \begin{align*}& \begin{aligned} Z_{cat}=cat\left(Z_{ad}^{1},Z_{ad}^{2}\right). \end{aligned}\end{align*}

And we introduce MLP to learn common features from multi-view scRNA-seq data. Thus, the common representation $Z_{final}$ of scRNA-seq data can be expressed as follows:


(27) \begin{align*}& \begin{aligned} Z_{final}=MLP\left(Z_{cat}\right). \end{aligned}\end{align*}

Finally, we employ the $k$-means algorithm to perform clustering operations on the fused features, and obtain the scRNA-seq data clustering results.

## Experiment

In this section, we perform several experiments on eight real scRNA-seq datasets to verify the superiority of the proposed method in clustering tasks.

### Evaluation Metrics

To make a fair comparison, we employed accuracy (ACC) [44], normalized mutual information (NMI) [45], adjusted rand index (ARI) [46], and fowlkes–mallows index (FMI) [47] to evaluate the clustering performance of the proposed method and its competitors. In general, the higher the values of these four metrics are, the closer the predicted label is to the true label, resulting in a better clustering effect.

### Datasets

To verify the effectiveness of the proposed method, we conducted clustering experiments on eight scRNA-seq datasets from different platforms. These datasets include the following: Klein [48], Young [49], Quake_Smart-seq2_Trachea (Trachea) [50], Quake_Smart-seq2_Diaphragm (Diaphragm) [50], and Quake_10x_Limb_Muscle (Muscle) [50], Quake_10x_Bladder (Bladder) [50], Pollen [51], Wang_Lung [52]. The details for each dataset are shown in Table 1.

**Table 1:** The details of eight real scRNA-seq datasets

**Datasets**	**Cells**	**Genes**	**Cell types**
Klein	2717	24047	4
Young	5685	25215	11
Trachea	1350	23341	4
Diaphragm	870	17973	5
Muscle	3909	16512	6
Bladder	2500	16867	4
Pollen	301	21721	11
Wang_Lung	9519	14561	2

### Parameter setting

The proposed method includes three important hyperparameters $\alpha $, $\beta $, and $\gamma $. Here, we set these three hyperparameters to 1.0, 1.0, and 0.5, respectively. Considering that too few layers would result in insufficient representational capacity, while having too many layers would lead to over-smoothing. Therefore, we empirically set the number of layers to 4. Furthermore, other relevant parameters in our proposed method were configured as follows: the learning rate was assigned a value of 0.01, and the number of MLP layers was determined to be 2. Additionally, the experimental environment is CUDA 11.6, Pytorch 1.13.1, Python 3.7, and the experimental equipment is Nvidia GeForce RTX 3090 GPU.

**Table 2:** The clustering results of different methods on the Klein, Young, Trachea, and Diaphragm datasets

	Klein				Young				Trachea				Diaphragm			
	ACC	NMI	ARI	FMI	ACC	NMI	ARI	FMI	ACC	NMI	ARI	FMI	ACC	NMI	ARI	FMI
$k$ -means	0.5616	0.4146	0.2961	0.5047	0.4216	0.2636	0.2089	0.3903	0.3422	0.1108	0.0034	0.3559	0.4896	0.2521	0.1580	0.4427
GraphSCC	0.8174	0.8147	0.7751	0.8419	0.4513	0.4802	0.3152	0.4123	0.6666	0.5479	0.4732	0.6714	0.7126	0.7507	0.6025	0.7393
sc-INDC	0.8747	**0.8198**	0.8215	0.8720	0.7078	0.7475	0.5968	0.6587	0.9278	0.8210	0.8679	0.9228	0.9741	0.9168	0.9573	0.9721
scMCKC	0.8334	0.7481	0.7675	0.8357	0.7778	0.7900	0.6757	0.7298	0.8986	0.7686	0.8501	0.9142	0.9849	0.9367	0.9624	0.9754
ADClust	0.8814	0.7021	0.7494	0.8204	0.6195	0.6301	0.5035	0.6343	0.7229	0.6229	0.6187	0.8140	0.9873	0.9455	**0.9744**	0.9833
scCDG	0.7066	0.7495	0.6412	0.7400	0.7141	0.6655	0.5876	0.6526	0.4674	0.4603	0.3007	0.5211	0.4908	0.5187	0.3598	0.5387
DeepScena	0.7927	0.6677	0.6604	0.7578	0.6856	0.6290	0.5622	0.6292	0.5066	0.2342	0.1601	0.5111	0.8091	0.6872	0.7464	0.8251
scMAE	0.9101	0.7736	0.8002	0.8557	0.8065	0.8109	0.7133	0.7583	0.6481	0.5046	0.3224	0.6214	0.9758	0.9184	0.9380	0.9603
CAKE	0.6235	0.4354	0.3822	0.6329	0.7284	0.7365	0.6164	0.6732	0.7000	0.6762	0.5566	0.7233	0.9230	0.8299	0.8715	0.9202
Louvain	0.8049	0.7866	0.7835	0.8435	0.7104	0.7915	0.6184	0.6928	0.3044	0.5329	0.1863	0.4072	0.8505	0.8337	0.7439	0.8298
scMMN	**0.9294**	0.8083	**0.8462**	**0.8888**	**0.8489**	**0.8198**	**0.7716**	**0.8085**	**0.9447**	**0.8382**	**0.8799**	**0.9327**	**0.9884**	**0.9496**	0.9457	**0.9841**

### Comparative experiment

To evaluate the effectiveness of the proposed method, we compared it with ten representative methods, such as $k$-means [53], GraphSCC [54], sc-INDC [23], scMCKC [24], ADClust [55], scCDG [56], DeepScena [57], scMAE [25], CAKE [26], and Louvain [58]. Since different datasets contain different numbers of clusters, we use different ways to determine the number of clusters for different comparison methods. For methods that can estimate the number of clusters themselves, such as ADClust, we do not set the number of clusters artificially. For methods that cannot estimate the number of clusters themselves, such as $k$-means, we set the number of clusters to the number of clusters in the real dataset. For each comparison method, on any dataset, all hyperparameters except for the number of clustering are fixed. Additionally, these hyperparameters are taken from the default parameters of the corresponding papers or code. A detailed description of each comparison method is included in Supplementary Methods.

**Table 3:** The clustering results of different methods on the Muscle, Bladder, Polle, and Wang_Lung datasets

	Muscle				Bladder				Pollen				Wang_Lung			
	ACC	NMI	ARI	FMI	ACC	NMI	ARI	FMI	ACC	NMI	ARI	FMI	ACC	NMI	ARI	FMI
$k$ -means	0.5466	0.4487	0.3768	0.5557	0.7544	0.5420	0.5375	0.7405	0.8571	0.9103	0.8489	0.8661	0.9831	0.8650	0.9275	0.9745
GraphSCC	0.7425	0.7958	0.7404	0.7979	0.6946	0.6543	0.6253	0.7762	0.7912	0.8842	0.7865	0.8174	0.9796	0.8367	0.9114	0.9699
sc-INDC	0.9492	0.9368	0.9374	0.9519	0.9953	0.9700	0.9871	0.9929	0.8946	0.9299	0.9133	0.9240	0.9307	0.6500	0.7266	0.8977
scMCKC	0.9253	0.9195	0.8763	0.9064	0.9972	0.9792	0.9915	0.9953	0.7774	0.8861	0.7621	0.8065	0.9311	0.6513	0.7281	0.8983
ADClust	0.9731	0.9217	0.9412	0.9459	0.7972	0.7361	0.7368	0.8475	0.8637	0.9168	0.8448	0.8708	0.6213	0.5386	0.3834	0.6213
scCDG	0.8055	0.8428	0.7446	0.8067	0.5352	0.6338	0.4269	0.6353	0.7109	0.7677	0.6413	0.6876	0.4350	0.4127	0.2105	0.5302
DeepScena	0.7759	0.8082	0.7772	0.8290	0.9716	0.9425	0.9807	0.9895	0.5880	0.5197	0.6327	0.6996	0.9738	0.7961	0.8881	0.9608
scMAE	0.9951	0.9798	0.9887	0.9913	0.9169	0.9328	0.9383	0.9451	0.9203	0.9306	0.9387	0.9455	0.9853	0.8629	0.9359	0.9778
CAKE	0.9775	0.9487	0.9565	0.9667	0.7740	0.7930	0.7149	0.8338	0.8605	0.9345	0.8508	0.8703	0.5227	0.4566	0.2708	0.5958
Louvain	0.5139	0.7363	0.4388	0.5801	0.3104	0.5377	0.2408	0.4731	0.8172	0.9058	0.7793	0.8083	0.2871	0.3376	0.1096	0.3914
scMMN	**0.9965**	**0.9856**	**0.9929**	**0.9946**	**0.9982**	**0.9872**	**0.9953**	**0.9974**	**0.9422**	**0.9515**	**0.9464**	**0.9523**	**0.9864**	**0.8714**	**0.9408**	**0.9795**

The experimental results of various scRNA-seq data clustering methods are shown in Tables 2–3. In these tables, the best value is highlighted in bold, and the second-best value is underlined. From the clustering results, we can draw the following conclusions:

(1) It can be seen that all methods demonstrate promising clustering results on the Diaphragm, Muscle, Bladder, Pollen, and Wang_Lung datasets, but fail to deliver satisfactory performances on the Klein, Young, and Trachea datasets. The main reason is that the latter three datasets contain more than 20000 genes. Meanwhile, most methods only extract 1000-3000 highly expressed genes from the gene expression matrix during pre-processing stage. These limited genes can negatively impact clustering accuracy, especially in datasets with a large number of genes. Interestingly, despite the Pollen dataset also containing over 20,000 genes, it performed well across all methods. This discrepancy can be attributed to the unique data distribution characteristics of the pollen dataset.
(2) Compared with other scRNA-seq data clustering methods, the proposed method achieves the highest values across four evaluation metrics on six scRNA-seq datasets. In the rest two datasets, such as Klein and Diaphragm, optimal values are achieved for three evaluation metrics. This unequivocally demonstrates the superiority of our method over other competitors in scRNA-seq data clustering problems.
(3) We can see that both sc-INDC and scMAE achieve the best average ACC among all competitors across eight scRNA-seq datasets. This is because these two methods can effectively address the inherent noise in scRNA-seq data. Specifically, scMAE employs a masked autoencoder to selectively mask genes, enabling the autoencoder to effectively learn the scRNA-seq cell representation and reconstruct the original data, yielding promising denoising results. sc-INDC focuses on maximizing the mutual information between input and output cell representations while minimizing the loss to preserve noise invariance. It can be observed that the clustering results of the ADClust and scMAKC methods are better than those of the DeepScena method. The main reason is that DeepScena exhibits significant advantages on large-scale scRNA-seq datasets. However, our experiments include datasets of different sizes, which results in a decrease in the performance of DeepScena on smaller datasets.
(4) The reason why our method can achieve better clustering performance compared with other methods lies in two points. The first point is that we adopt a multi-view and multi-level cell feature learning method, which enables the model to fully learn the latent representation of cells. The second point is that we introduce the structural consistency contrast constraint, which guides the model to train from the perspective of cell structure. In summary, since learning single-cell data mainly starts from two aspects, representation and structure. We design the model from the aspects of structure and gene representation respectively, so we get better experimental results.

**Table 4:** The ablation experiment on the Young dataset

	**ACC**	**NMI**	**ARI**	**FMI**
w/o G	0.5932	0.4960	0.4483	0.5371
w/o F	0.7928	0.7652	0.6843	0.7338
w/o S	0.8443	0.8112	0.7517	0.7915
w/o L	0.8454	0.8140	0.7560	0.7949
w/o M	0.7850	0.7709	0.6735	0.7244
scMMN	**0.8489**	**0.8198**	**0.7716**	**0.8085**

**Table 5:** The ablation experiment on the Muscle dataset

	**ACC**	**NMI**	**ARI**	**FMI**
w/o G	0.8337	0.7369	0.7872	0.8390
w/o F	0.9898	0.9688	0.9825	0.9866
w/o S	0.8644	0.8092	0.7527	0.8087
w/o L	0.9906	0.9697	0.9843	0.9880
w/o M	0.9842	0.9544	0.9737	0.9798
scMMN	**0.9965**	**0.9856**	**0.9929**	**0.9946**

**Table 6:** The ablation experiment on the Pollen dataset

	**ACC**	**NMI**	**ARI**	**FMI**
w/o G	0.9352	0.9447	0.9268	0.9349
w/o F	0.9289	0.9448	0.9268	0.9401
w/o S	0.9036	0.9231	0.9324	0.9082
w/o L	0.9289	0.9468	0.9399	0.9467
w/o M	0.9302	0.9514	0.8969	0.9484
scMMN	**0.9422**	**0.9515**	**0.9464**	**0.9523**

### Analysis of simulated data

To verify the performance and generalization of the proposed method under controlled conditions, we generate a simulated scRNA-seq dataset via Splatter [59] to compare with other methods. Here, the parameters of the simulated dataset were set as follows: 5000 cells, 5000 genes, and dropout values of -1.0, -0.5, 0, 0.5. Supplementary Figure S1 shows the clustering results of various methods on the simulated scRNA-seq dataset under different dropout settings. It can be seen from the figure, the performance of each method decreases as dropout increases, illustrating the greater the challenge of single-cell clustering as noise increases. Our method achieves optimal values in each dropout case, which indicates that our method has good generalization under controlled conditions.

**Figure 2:**
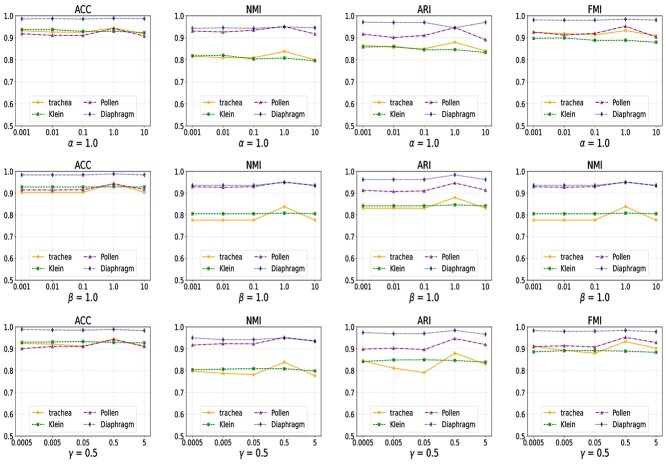
Experimental results of parameter sensitivity on the Klein, Trachea, Diaphragm, and Pollen datasets.

### Analysis of small clusters

Considering that real scRNA-seq datasets contain many small clusters, accurate clustering of small cells is very important. So in this part, we verify the clustering performance of the proposed method on small clusters. Supplementary Table S1 shows the number of clusters contained in each datasets. Supplementary Table S2 and Supplementary Table S3 shows the clustering results of different methods on the small clusters. It can be found from Supplementary Table S2 and S3 that the proposed method achieve the highest value on every small cluster, indicating that our method is also suitable for small cluster identification and clustering.

### Computational complexity

In this part, we calculate the the parameters (Params) and floating-point operations (FLOPs) of each deep learning-based method, and the experimental results are shown in Supplementary Table S4. From the table, it can be seen that our method has a small computational complexity and parameter number. This is due to the fact that our main framework is only two-layer GCNs and MLP is used in the feature fusion part, both of which have less complexity.

### Ablation study

To verify the effectiveness of each module in our proposed method, we conducted ablation experiments on the Diaphragm, Muscle, and Pollen datasets. Specifically, we replaced the GCNs in our method with MLP to verify the effectiveness of the representation learning module. The symbol of this variant is denoted by w/o G. Secondly, we performed the ablation study on the feature fusion module. Thus, this variant is constructed by removing the MLP from the feature fusion module. It can be symbolically represented as w/o F. To show the effectiveness of the structural consistency contrastive learning module, we removed this module and denoted it as w/o S. Finally, to prove the contributions of GLFs, we varied the stacked layers of GLFs from 1 to 4, which is denoted by w/o L. Considering that the proposed model belongs to a multi-view clustering approach, we changed the model from multiple views to a single view, denoted as w/o M. The experimental results on the Young, Muscle, and Pollen datasets are shown in Table 4–6.

Experiments have shown that our proposed scMMN method outperforms other variants on these three datasets. It is clear to see that the average accuracy decreases significantly when a module is replaced or removed. Specifically, a decrease of 14.18$\%$ in average accuracy can be observed after replacing GCNs with MLP. Furthermore, the removal of the feature fusion module results in a more moderate but still significant decrease of 2.54$\%$ in average accuracy. Upon discarding the structural consistency constraint, the average ACC decreases by 5.84$\%$. Finally, the conversion from multi-view to single-view reduces the average ACC by 2.94$\%$. However, we can see that after eliminating each module, our algorithm has a significant performance impact on the Young dataset, while the Pollen dataset is less affected. This difference between these datasets can be attributed to their distinct data distributions.

**Figure 3:**
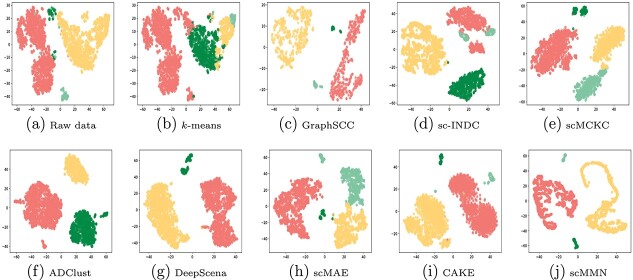
Clustering results of different methods on the Bladder dataset.

**Figure 4:**
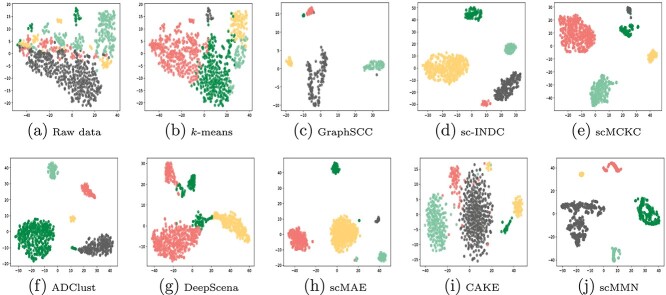
Clustering results of different methods on the Diaphragm dataset.

### Parameter sensitivity analysis

The proposed method involves three hyperparameters $\alpha $, $\beta $, and $\gamma $. In this section, we conducted several experiments on the Klein, Trachea, Diaphragm, and Pollen datasets to verify their sensitivity. The experimental results of the proposed method with different hyperparameter settings are shown in Fig. 2. The parameters $\alpha $ and $\beta $ were set to 0.001, 0.01, 0.1, 1.0, and 10.0, respectively. The parameter $\gamma $ was set to 0.0005, 0.005, 0.05, 0.5, and 5, respectively.

It can be seen from the experimental results that the clustering performance of the proposed model undergoes slight changes with variations in parameter values. However, the performance of the proposed method on the Pollen dataset exhibits the most stable clustering performances, showing minimal variation with changes in parameter values. Although the performances on the Klein and Diaphragm datasets exhibit slight under different parameter settings, none of them exceed 0.05. In addition, the clustering performance on the Trachea dataset shows significant visual fluctuations with a maximum variation of 0.1, but it remains within the acceptable range. In summary, it can be inferred that our proposed method is relatively insensitive to the hyperparameters of the proposed model.

### Visualization

In this subsection, we visualized the results of all methods to demonstrate the advantage of the proposed method. Here, we employed the $t$-SNE method to draw the distribution of scRNA-seq data. The visualization results on the Bladder and Diaphragm datasets are shown in Figures 3-4.

These experimental results clearly illustrate the differences between traditional clustering algorithms and deep learning algorithms in cell partitioning within different clusters. The former displays unclear boundaries between clusters, whereas the latter exhibits distinct separation among different clusters. In addition, GraphSCC, scCMCK, and ADClust stand out among the deep learning methods for their ability to accurately distinguish between clusters with significant intervals. Compared with other competitors, our scMMN method shows the best clustering results on the Bladder and Diaphragm datasets. It can accurately distinguish clusters without any overlap, demonstrating its excellent clustering performance.

## Discussion and conclusion

In this paper, we propose a novel scRNA-seq data clustering method, termed multi-level multi-view network based on structural consistency contrastive clustering (scMMN). Unlike existing scRNA-seq data clustering methods,our method clusters scRNA-seq data from the perspective of multi-level and multi-view. Specifically, it first constructs the shallow and deep views of scRNA-seq data, thus offering a comprehensive representation. In the feature fusion model, our scMMN method uses MLP to fuse the features and thus learn a common representation of different views. In addition, this method constructs group contrastive loss functions for features and a structural consistency contrastive loss function for structural relationships to guide the model training. Experimental results show that our proposed scMMN method outperforms other competitors in scRNA-seq data clustering applications.

Although our proposed method has achieved satisfactory clustering results, several issues still need to be addressed in future work. For example, our method can only cluster from the perspective of the gene expression matrix. In the future, we aim to design models that integrate gene regulatory relationships, gene functions, and other factors to enhance the performance of scRNA-seq data clustering.

**Key Points**

- We propose a novel multi-level multi-view contrastive clustering framework for clustering scRNA-seq data. The framework uses a general method to construct the adjacency matrix of cells, called the shallow view, and uses the graph Laplacian filter to construct the deep view.
- Multi-view features and structural contrastive learning strategies are introduced to learn common features and explore the structural relationship between cells.
- We adopted a group learning strategy to supervise the model to learn cell representations at different levels of the cell.
- The proposed method can accurately learn the latent representation of cells, which is very beneficial for the subsequent representation learning and clustering process.
- We conduct comprehensive experiments on eight scRNA-seq datasets, and scMMN can obtain good clustering results. It shows that our method has good biological effect.

## Supplementary Material

Supplementary_material_bbae562
