# Supplementary material for: Multi-level multi-view network based on structural contrastive learning for scRNA-seq data clustering
Source: Brief Bioinform. 2024 Nov 4;25(6):bbae562. doi: 10.1093/bib/bbae562 (PMC11532661; doi:10.1093/bib/bbae562)
Supplement: Supplementary_material_bbae562 [file supplementary_material_bbae562.docx]

**Supplementary Materials — “Multi-level multi-view network based on structural contrastive learning for scRNA-seq data clustering”**

Zhenqiu Shu^1^, Min Xia^1^, Kaiwen Tan^1,∗^, Yongbing Zhang^1^ and Zhengtao Yu^1^

^1^Faculty of Information Engineering and Automation, Kunming University of Science and Technology, Chenggong, 650500, Yunnan, China

^∗^Corresponding author. kwtan0909@qq.com

**Supplementary Figure S1**. The clustering result of various methods on the simulated data.


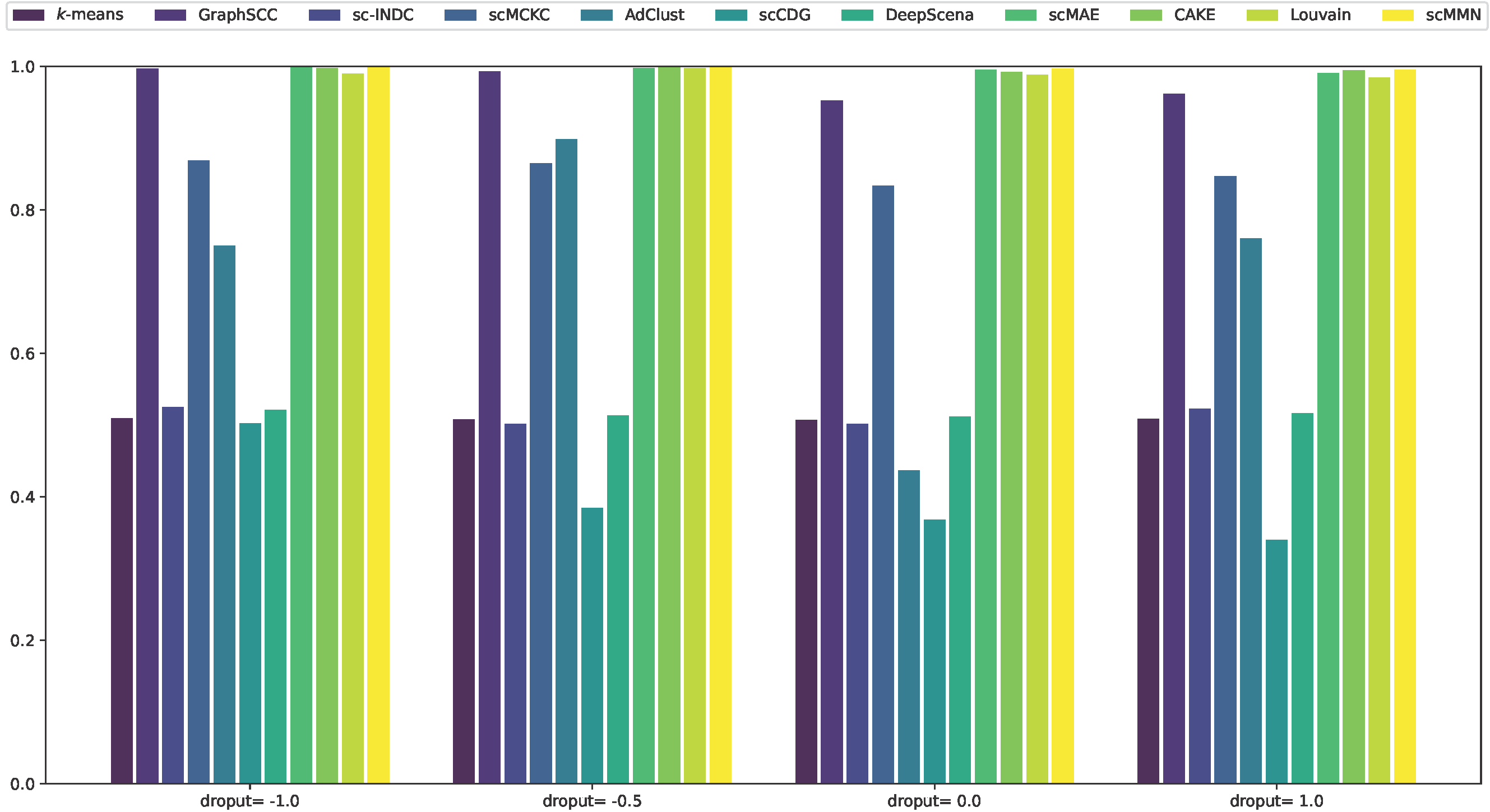


1. ACC


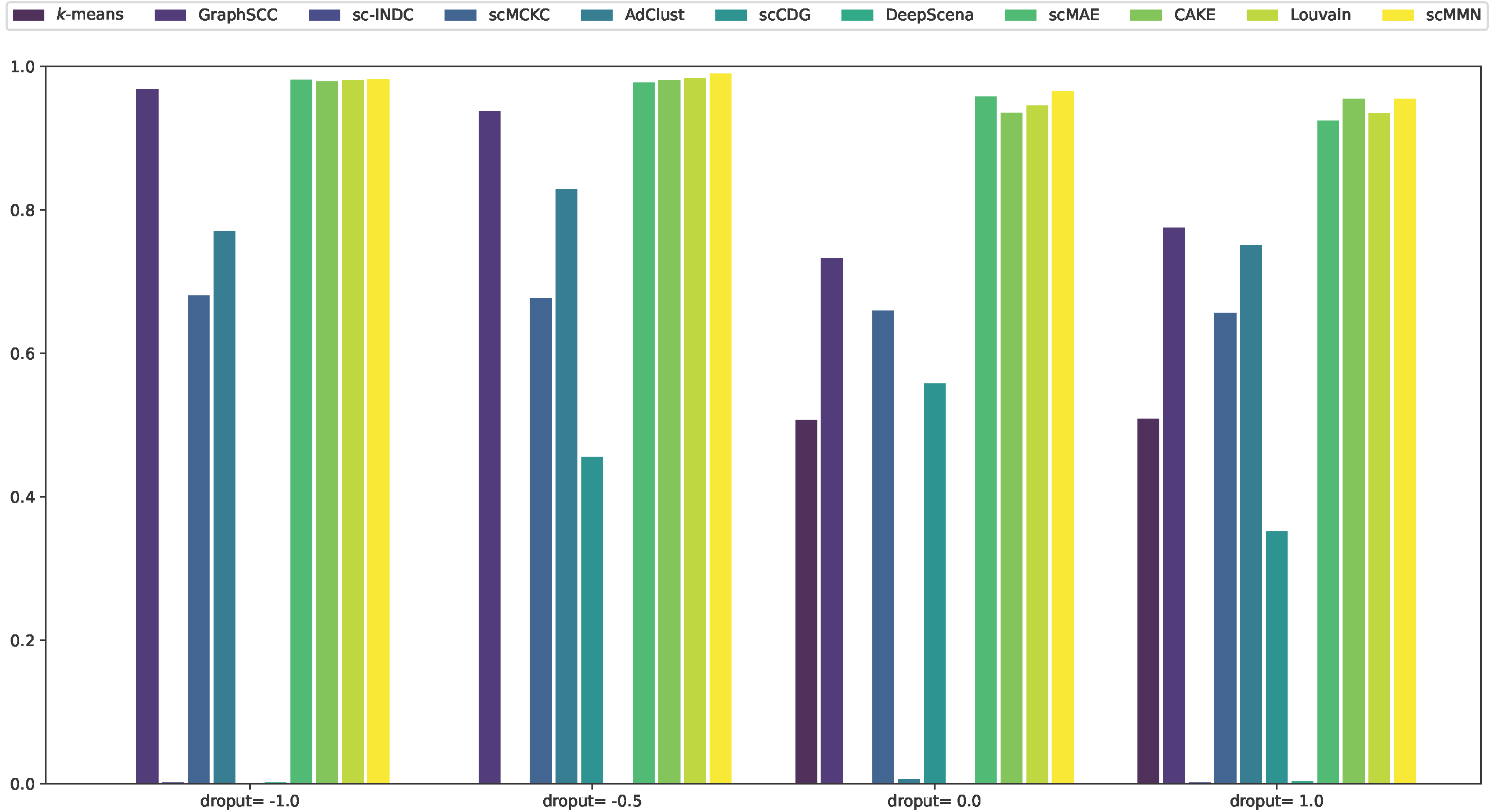


1. NMI


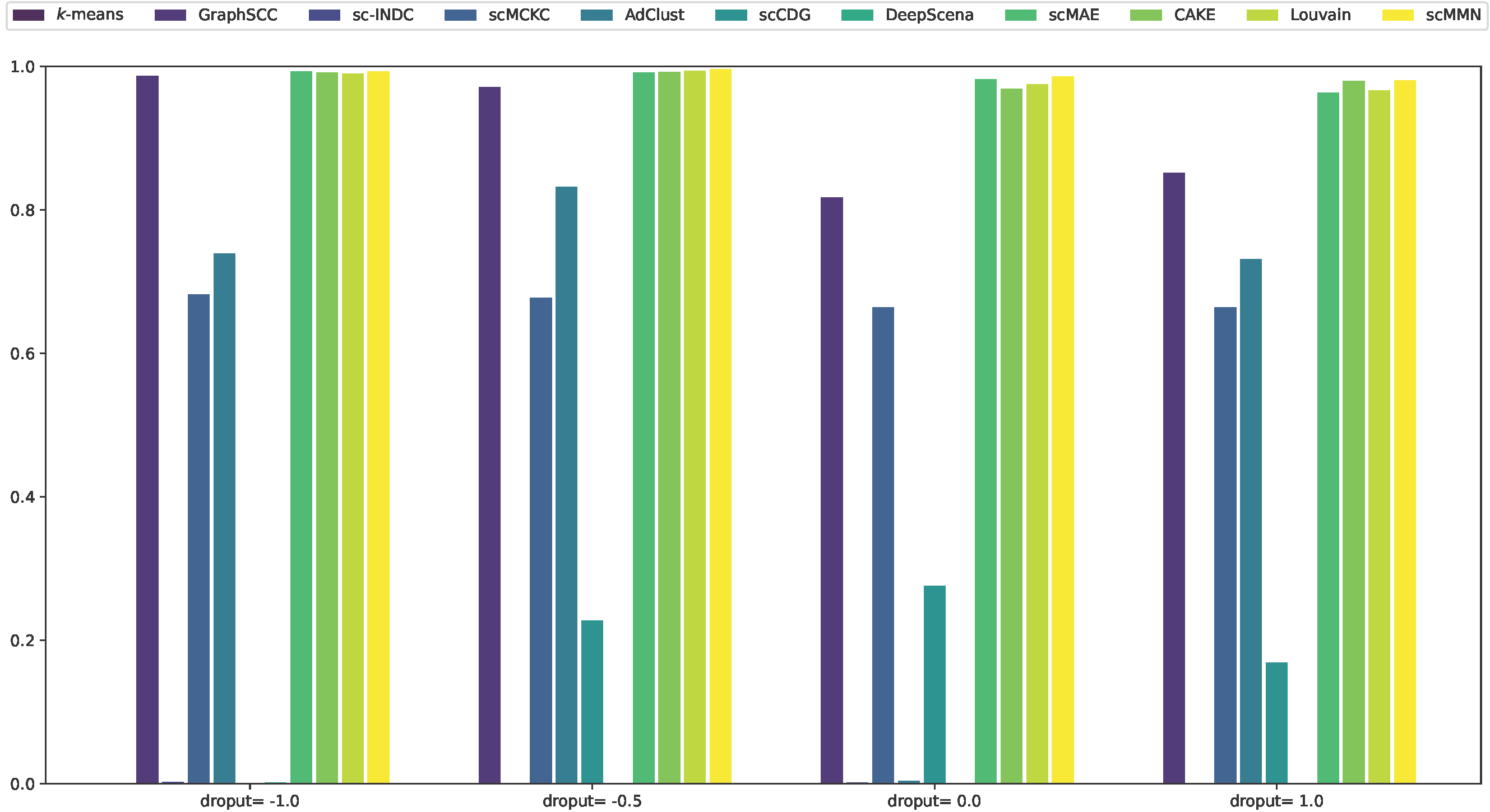


1. ARI


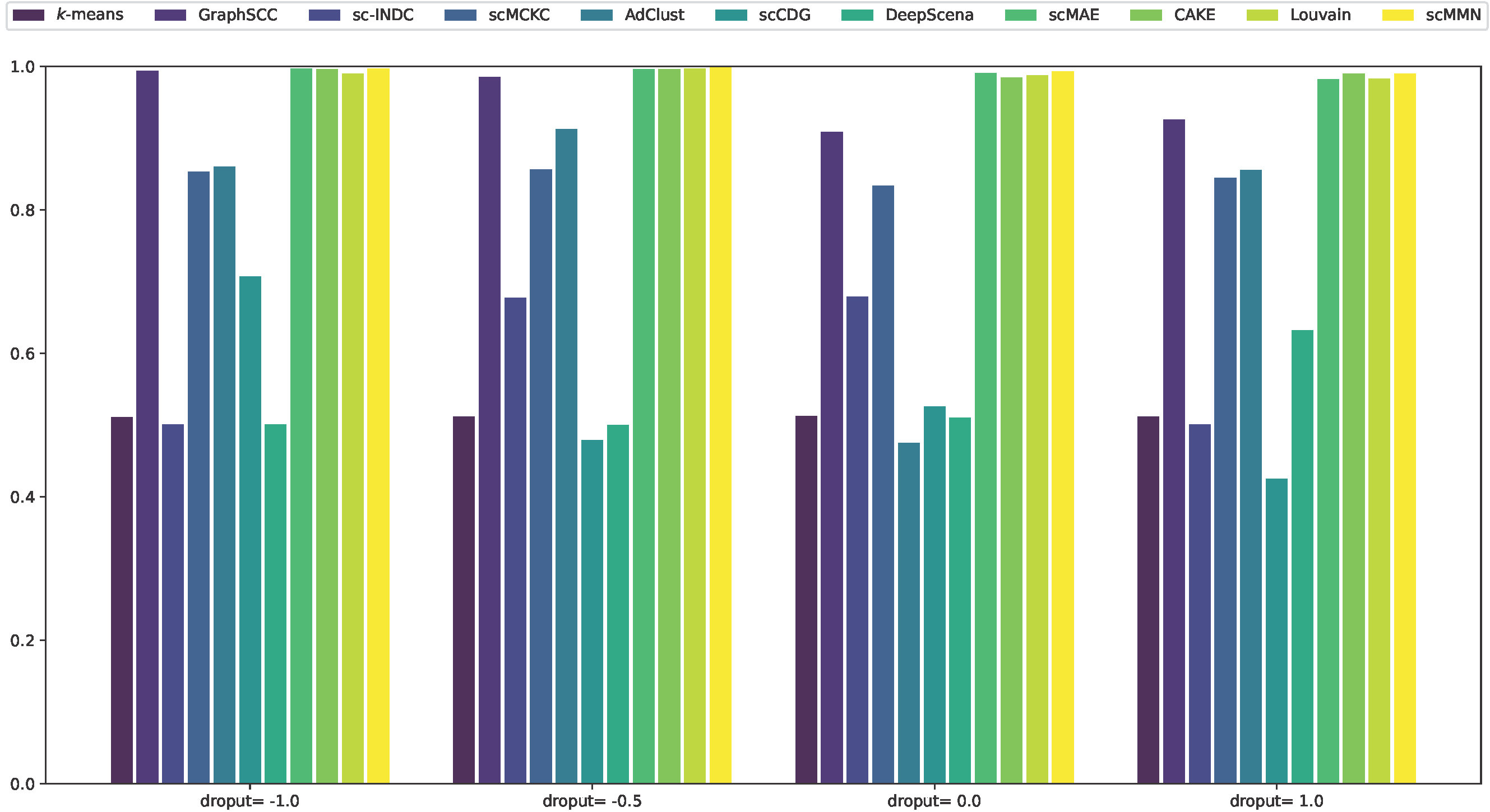


1. FMI

**Supplementary Table S1.** The number of clusters contained in each datasets.


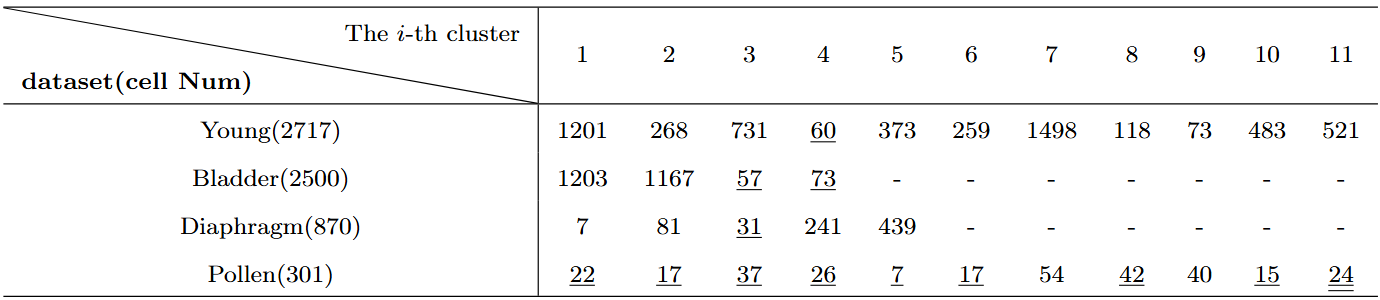


**Supplementary Table S2.** The clustering results of different methods on 7 small clusters.


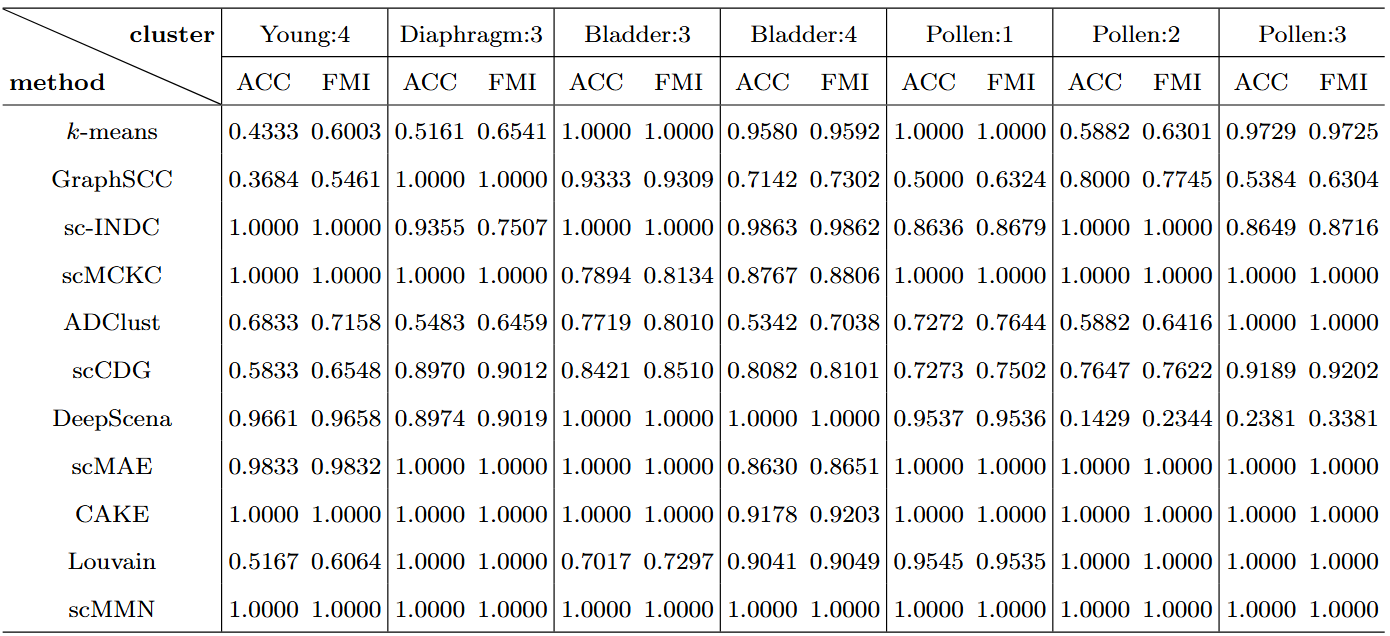


**Supplementary Table S3.** The clustering results of different methods on 8 small clusters.


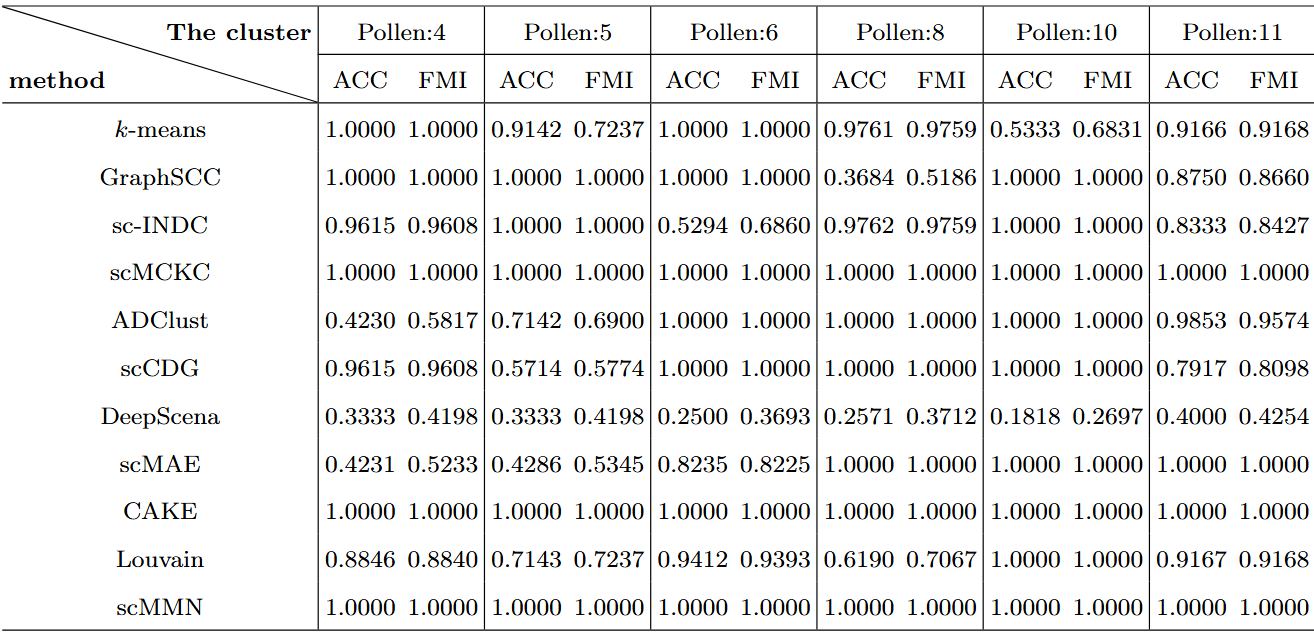


**Supplementary Table S4.** The computational complexity of various deep learning-based methods.


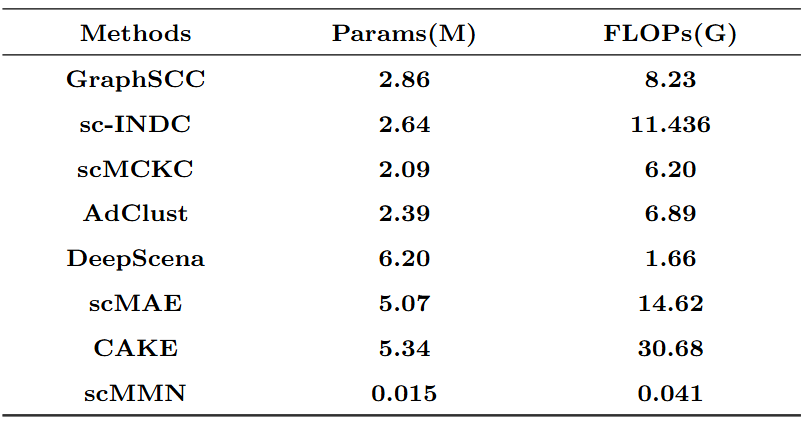


**Supplementary Methods**

**Detailed information of clustering algorithms**

1. k-means: It is a baseline clustering algorithm in scRNA-seq data clustering tasks. The code of k-means is available at https://scikit-learn.org/dev/modules/clustering.html#k-means.
2. GraphSCC: It adopts GCNs to learn potential representations and similarities of cells, and then constructs a dual self-supervised module to supervise model training. Its code is available at https://github.com/biomed-AI/GraphSCC.
3. sc-INDC: This method seeks to address the issue of high noise in scRNA-seq data. The code of sc-INDC is available at https://github.com/arnabkmondal/sc-INDC.
4. scMCKC: This method is an autoencoder framework based on the ZINB model, which introduces a novel cell compactness constraint to guide the model training. The code of scMCKC is available at https://github.com/leaf233/scMCKC.
5. ADClust: It is an automatic deep latent feature learning method. Its advantage lies in its ability to automatically determine the number of clusters. The code of ADClust is available at https://github.com/biomed-AI/ADClust.
6. scCDG: This method employs autoencoders to remove noise and subsequently utilizes GCNs to learn the latent representation of cells. The code of scCDG is available at https://github.com/WHY-17/scCDG.
7. DeepScena: This method is a deep autoencoder framework that introduces hierarchical learning and selfsupervised learning. The code of DeepScena is available at https://github.com/shaoqiangzhang/DeepScena.
8. scMAE: This is a scRNA-seq data clustering method based on the mask autoencoder. The code of scMAE is available at https://github.com/CSUBioGroup/scMAE.
9. CAKE: This method is a self-supervised model based on a self-knowledge distiller. Its code can be found at https://github.com/CSUBioGroup/CAKE.
10. Louvain: It is a classic community discovery algorithm. During our comparative experiment involving this method, we adopted a similar preprocessing method before data clustering. The source code of Louvain is available at: http://www.nature.com/reprints/index.html.
